# Temporal Stability of Smartphone Use Data: Determining Fundamental Time Unit and Independent Cycle

**DOI:** 10.2196/12171

**Published:** 2019-03-26

**Authors:** Yuan-Chien Pan, Hsiao-Han Lin, Yu-Chuan Chiu, Sheng-Hsuan Lin, Yu-Hsuan Lin

**Affiliations:** 1 National Health Research Institutes Institute of Population Health Sciences Miaoli County Taiwan; 2 National Taiwan University Department of Psychology Taipei Taiwan; 3 MacKay Memorial Hospital Department of Psychiatry Taipei Taiwan; 4 National Chiao Tung University Institute of Statistics Hsinchu Taiwan; 5 National Taiwan University Hospital Department of Psychiatry Taipei Taiwan; 6 National Taiwan University Department of Psychiatry, College of Medicine Taipei Taiwan; 7 National Taiwan University Institute of Health Behaviors and Community Sciences, College of Public Health Taipei Taiwan

**Keywords:** temporal stability, smartphone use, smartphone addiction, smartphone, mobile phone

## Abstract

**Background:**

Assessing human behaviors via smartphone for monitoring the pattern of daily behaviors has become a crucial issue in this century. Thus, a more accurate and structured methodology is needed for smartphone use research.

**Objective:**

The study aimed to investigate the duration of data collection needed to establish a reliable pattern of use, how long a smartphone use cycle could perpetuate by assessing maximum time intervals between 2 smartphone periods, and to validate smartphone use and use/nonuse reciprocity parameters.

**Methods:**

Using the Know Addiction database, we selected 33 participants and passively recorded their smartphone usage patterns for at least 8 weeks. We generated 4 parameters on the basis of smartphone use episodes, including total use frequency, total use duration, proactive use frequency, and proactive use duration. A total of 3 additional parameters (root mean square of successive differences, Control Index, and Similarity Index) were calculated to reflect impaired control and compulsive use.

**Results:**

Our findings included (1) proactive use duration correlated with subjective smartphone addiction scores, (2) a 2-week period of data collection is required to infer a 2-month period of smartphone use, and (3) smartphone use cycles with a time gap of 4 weeks between them are highly likely independent cycles.

**Conclusions:**

This study validated temporal stability for smartphone use patterns recorded by a mobile app. The results may provide researchers an opportunity to investigate human behaviors with more structured methods.

## Introduction

### Background

The excessive use of smartphones has become a substantial worldwide social issue because of increasing smartphone penetration. Recording human behaviors (eg, smartphone use, exercise, and sleep time) via a smartphone is a feasible and popular method in modern society. A previous study has found that smartphone use patterns can reflect social economic status in Rwanda [1]. Given the convenience of smartphones, health-related mobile apps might serve as a “digital lifeline,” particularly in rural and low-income regions, helping mental health care professionals with medical intervention and behavioral modification [2]. Using smartphone use data to assess human behaviors and to assist monitoring pattern of behaviors has become a crucial issue in this century.

For the assessment of smartphone use patterns, we introduced several app-generated parameters to delineate smartphone usage. There are 2 fundamental app-generated parameters about smartphone use, namely use frequency and use duration. To assess core elements about Web-based behaviors, we also developed 2 parameters—root mean square of successive differences (RMSSD) and Similarity Index (SI)—to assess the reciprocity between use and nonuse patterns. We calculated the RMSSD and SI within a day and applied the average daily RMSSD and SI to indicate impaired control and compulsive behaviors for smartphone use [3]. Furthermore, previous studies claimed that self-reported problematic smartphone use did not correlate with actual use recorded by an app [4,5]. It implies that problematic smartphone use pattern may not be captured through self-reported scales. However, we have found that proactive use may be more representative to addictive behavior than total use. To investigate the relationship between smartphone use behaviors and self-reported problematic smartphone use, this study also conducted an exploratory analysis of associations between app-generated parameters and smartphone addiction symptoms.

However, frequent short-period smartphone use is difficult to measure with either self-reporting or the reporting of others. Thus, an app that automatically detects smartphone use is likely a more reliable assessment tool with ecological validity as it can record smartphone use behaviors in a naturalistic setting. Few studies have used mobile apps to measure smartphone use behaviors directly [4,6,7]. The total duration of data collection varies in different studies, ranging from 1 to 6 weeks [4,7,8]. A previous study demonstrated that a relatively short duration of behavioral data is required to qualify a 2-week use period [5]. However, even a 1-month record might not be enough to allow detection for patterns of smartphone use [3]. For an app-generated parameter, it may need a detection time period of longer than 1 month. Our previous study also found that smartphone use behaviors demonstrate a weekly cycle [7]. In addition, previous studies did not discriminate active smartphone use (ie, proactive use) from smartphone use triggered by a notification (ie, reactive use) [4,5]. It may be crucial to separate proactive smartphone use from total smartphone use to make a more reliable inference for actual use in natural setting. To determine the shortest duration of smartphone use, data are required to reliably infer a pattern of smartphone use, validating long-term temporal stability for daily smartphone usage, and examining more app-generated parameters except frequency and duration are urgently needed.

### Objective

The specific aims of this study were to (1) illustrate the time periods or span of weeks required to reliably infer patterns of long-term smartphone use, (2) investigate how long could a smartphone use cycle perpetuate by assessing maximum time intervals (TIs, ie, weeks) between 2 smartphone use periods, and (3) validate smartphone use and use/nonuse reciprocity parameters.

## Methods

### Participants and Procedure

The “Know Addiction” database collected smartphone use data from March 2017 to March 2018. We selected 33 healthy adult participants (28 men, mean age 29.48, SD 10.44 years, range: 18-62) who had smartphone use data for at least 8 weeks. Data collected on the first day and the last day were excluded because of the incomplete nature. We used a 5-item questionnaire to assess smartphone addiction. This study was approved by the institutional review board of National Health Research Institutes, who waived the need for written informed consent as the data were analyzed anonymously.

### Measures

#### The App-Generated Parameters

We defined an episode of smartphone use as a time period from screen-on to the successive screen-off. Know Addiction calculated daily episode count as total use frequency (F). Similarly, the total daily episode lengths were calculated as total use duration (D). Next, we distinguished “proactive use” from “reactive use.” A proactive use was defined as 1 use episode without any notification within 1 min before the screen-on. It is conceivable that proactive use may be more representative of addictive behavior and total use [3]. We calculated daily episode counts and total length of proactive use as the proactive use frequency (PF) and the proactive use duration (PD), respectively.

**Figure 1:**
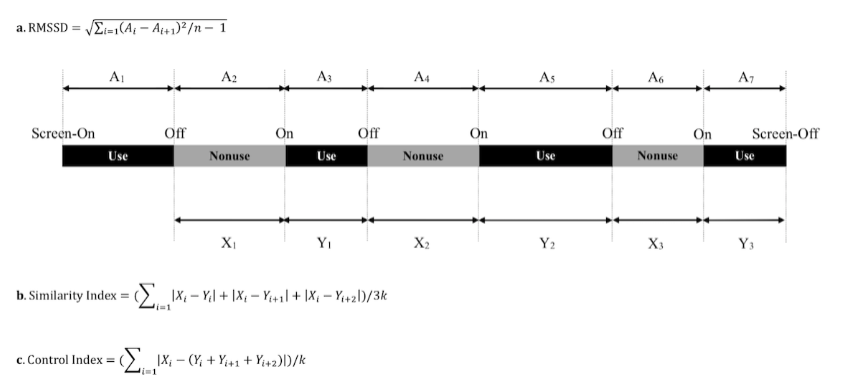
Use/nonuse reciprocity parameters. (a) A schematic and the equation used to calculate the root mean square of the successive differences (RMSSD). Ai is the duration of a use or nonuse epoch. There are (n-1) successive differences of use/nonuse episodes. The RMSSD was calculated to be the square root of the mean square. (b) A schematic and the equation used to calculate the Similarity Index (SI), Xi refers to the duration of a nonuse (gray) episode, Yi refers to the duration of a use (black) episode. Each nonuse episode (Xi) corresponds to 3 successive use epochs (Yi, Yi+1, and Yi+2). k is the number of nonuse episodes in a day. Thus, the SI is the average absolute difference between the nonuse and use episodes. (c) A schematic and the equation used to calculate the Control Index (CI), Xi refers to the duration of a nonuse (gray) episode and Yi refers to the duration of a use (black) episode. Each nonuse episode (Xi) corresponds to 3 successive use epochs (Yi, Yi+1, and Yi+2). k is the number of nonuse episodes in a day. Thus, the CI is the average of the absolute differences between 1 nonuse episode and the total of the following 3 use episodes. RMSSD: root mean square of the successive differences.

We developed 3 parameters to delineate the reciprocity between the use and nonuse patterns in our previous study, namely RMSSD, SI [3], and an updated version of SI, Control Index (CI). Figure 1 shows the algorithm of the RMSSD. First, we calculated the difference between the adjacent duration of use (*A*_*i*_) and nonuse episodes (*A*_*i+1*_). Sleeping time was excluded from the nonuse episode. Next, each use/nonuse difference was passed through a sum of the squares and divided by (*n*-1) number of episodes. Finally, the RMSSD was calculated to be the square root of the mean square [3].

Figure 1 also shows the algorithm of the SI and CI. We calculated the absolute differences between 1 nonuse episode (*X*_*i*_) and its corresponding 3 successive use episodes (*Y*_*i*_, *Y*_*i+1*_, and *Y*_*i+2*_). The SI was calculated to be the average of the absolute differences between 1 nonuse episode and the nonuse and use episodes within a day. The CI was calculated to be the average of the absolute differences between 1 nonuse episode and the total of the following 3 use episodes.

In our previous study, we delineated the compulsive smartphone use parameters by 1 nonuse episode corresponded to 3 successive use episodes. This is because the use/nonuse parameter (ie, SI) was the most consistent parameter with psychiatrists’ clinical diagnosis [9]. The CI is an updated version of the SI, which may be a more representative index for the control ability of smartphone use.

A lower RMSSD indicates a lower variability and a higher similarity. However, RMSSD delineates only the reciprocity of the adjacent use and nonuse episodes. To demonstrate a more generalized form of use/nonuse reciprocity, we proposed the SI and the CI to investigate the craving to use the smartphone by assessing the reciprocity of 1 nonuse episode with its upcoming 3-use episodes. In this study, the temporal stability of smartphone use parameters (ie, F, D, PF, and PD) and use/nonuse parameters (ie, RMSSD, SI, and CI) was both examined.

#### The 5-Item Smartphone Addiction Inventory

The 5-item Smartphone Addiction Inventory (SPAI-5) is a 5-item version of the SPAI. The original SPAI is a 26-item self-reported inventory [10]. Participants were asked to rate items on a 4-point Likert scale ranging from 1 (strongly disagree) to 4 (strongly agree). The SPAI demonstrated very good internal consistency (Cronbach alpha=.94).

#### Statistical Analysis

**Figure 2:**
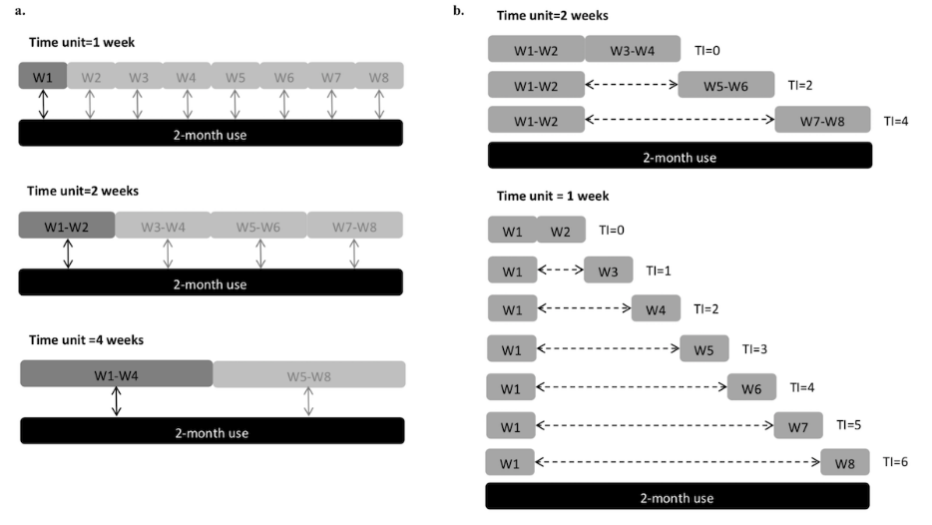
Illustration of validating the temporal stability of app-generated parameters. (a) Fundamental time unit. We chose 1 week, 2 weeks, and 4 weeks as the fundamental time units. The correlations among different time units and the 2-month use were analyzed. (b) Independent cycle. We determined independent cycle of smartphone use by calculating the correlations of 2 independent use periods with different time intervals. W1, W2,...W8: Week 1, Week 2,...Week 8; TI: Time interval (week).

Pearson correlation coefficient was used to demonstrate the relationships between app-generated parameters and the total score of SPAI-5. To illustrate the minimum number of weeks required to reliably infer patterns of smartphone use for a 2-month period, we chose 1 week, 2 weeks, and 4 weeks as the fundamental time units. The average daily app-generated parameters within the 2-month period were used. The correlations among different time units and the 2-month use were analyzed to provide an indication of how well a time unit is representative of a typical 2-month’s use (Figure 2). We adopted .75 as a criterion of “high correlation” for evaluating the efficiency of these time units.

Therefore, we investigated how long a smartphone use cycle could perpetuate by assessing maximum TIs between 2 smartphone periods for determining an independent cycle of smartphone use (Figure 2). For example, when the time unit used is 1 week, we first calculate the Pearson correlation coefficients between adjacent weeks (eg, week 1 vs week 2). A total of 7 correlation coefficients were calculated and then averaged to form a general index of temporal reliability for TI equal to 0. Next, the index for TI equals 1 (eg, week 1 vs week 3) was also calculated. Finally, the index for TI range 6 (ie, week 1 vs week 8) was calculated.

## Results

### The Correlations Between App-Generated Parameters and Smartphone Addiction

Table 1 shows the descriptive statistics and correlation coefficients between app-generated parameters and the total scores of SPAI-5 within 2 months. The proactive use frequency (mean 22.97, SD 14.11) accounts for 40% of the total use frequency (mean 57.29, SD 22.96). The proactive use duration (mean 4806.66, SD 10279.38) accounts for 23% of the total use duration (mean 20666.96, SD 12702.38). There was a significant correlation between proactive use duration and smartphone addiction (*r*=.40, *P*=.02).

### The Temporal Stability of App-Generated Parameters

**Figure 3:**
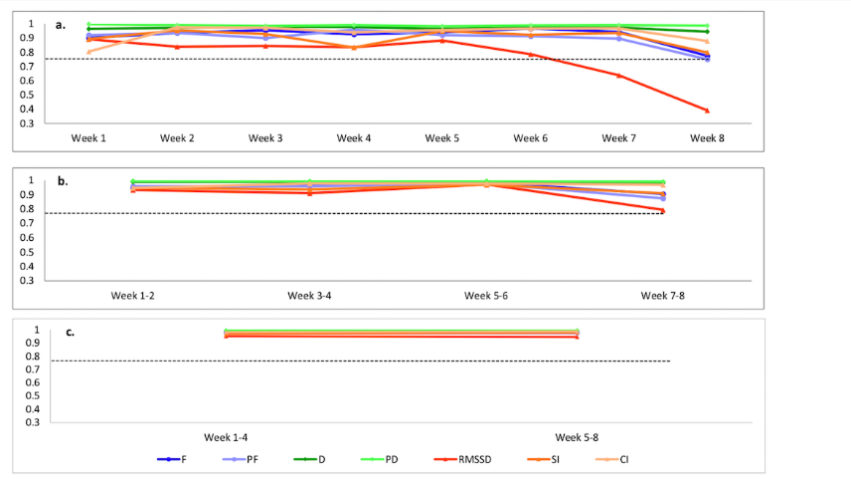
The temporal stability of app-generated parameters: Fundamental time unit. (a) The correlation coefficients between 1-week use and 2-month use for app-generated parameters. (b) The correlation coefficients between 2-week use and 2-month use for app-generated parameters. (c) The correlation coefficients between 4-week use and 2-month use for app-generated parameters. CI: Control Index; F: Total use frequency; D: Total use duration; PD: Proactive use duration; PF: Proactive use frequency; RMSSD: root mean square of the successive differences (between the adjacent duration of use and nonuse episodes); SI: Similarity Index.

To determine the shortest duration of smartphone use data required to reliably infer a pattern of long-term smartphone use, we calculated correlations for app-generated parameters between 3 time units (ie, 1 week, 2 weeks, and 4 weeks) and a 2-month period. Figure 3 shows the correlation coefficients between 1-week use and 2-month use for app-generated parameters. For 7 parameters, all correlations are statistically significant and above .75 from week 1 to week 6. There is a decrease for correlations of RMSSD after week 6 (week 7: *r*=.64, *P*<.001; week 8: *r*=.39, *P*=.03). The correlations of use frequency, proactive use frequency, and SI also show a decreased trend at week 8.

The correlation coefficients between 2-week use and 2-month use for app-generated parameters are shown in Figure 3. All correlations are statistically significant and above .75 between week 1 to 2 and week 7 to 8. The correlation coefficients between 4-week use and 2-month use for app-generated parameters are also shown in Figure 3. The correlations are statistically significant and above .90 between week 1 to 4 and week 5 to 8. The averages of correlations for app-generated parameters between 3 time units and 2-month use are summarized in Table 2.

**Table 1:** Means, SDs, and correlations of app-generated parameters, and the total score of the 5-item Smartphone Addiction Inventory.

Number	Parameters	Mean (SD)	1	2	3	4	5	6	7
1	F^a^	57.29 (22.96)	—^b^	—	—	—	—	—	—
2	D^c^ (second)	20666.96 (12702.38)	−.078	—	—	—	—	—	—
3	PF^d^	22.97 (14.11)	.688^e^	−.103	—	—	—	—	—
4	PD^f^ (second)	4806.66 (10279.38)	−.218	.551^e^	.180	—	—	—	—
5	RMSSD^g^	3070.41 (1421.50)	−.724^e^	.140	−.428^h^	.356^h^	—	—	—
6	SI^i^	1417.35 (853.55)	−.803^e^	.164	−.466^e^	.368^h^	.934^e^	—	—
7	CI^j^	1949.38 (1492.87)	−.666^e^	.608^e^	−.347^h^	.727^e^	.777^e^	.838^e^	—
8	SPAI-5^k^	12.55 (2.41)	−.089	.297	−.136	.403^h^	.139	.099	.314

^a^F: total use frequency.

^b^—:not applicable.

^c^D: total use duration.

^d^PF: proactive use frequency.

^e^*P*<.01.

^f^PD: proactive use duration.

^g^RMSSD: root mean square of the successive differences (between the adjacent duration of use and nonuse episodes).

^h^*P*<.05.

^i^SI: Similarity Index.

^j^CI: Control Index.

^k^SPAI-5: 5-item Smartphone Addiction Inventory.

**Table 2:** The averages of correlations for app-generated parameters between 3 time intervals and 2-month use.

Number	Parameters	1 week	2 weeks	4 weeks
1	F^a^	.918	.952	.979
2	D^b^	.969	.988	.993
3	PF^c^	.900	.943	.975
4	PD^d^	.990	.996	.998
5	RMSSD^e^	.764	.903	.950
6	SI^f^	.902	.942	.975
7	CI^g^	.933	.969	.986

^a^F: total use frequency.

^b^D: total use duration.

^c^PF: proactive use frequency.

^d^PD: proactive use duration.

^e^RMSSD: Root mean square of successive differences (between the adjacent duration of use and nonuse episodes).

^f^SI: Similarity Index.

^g^CI: Control Index.

**Figure 4:**
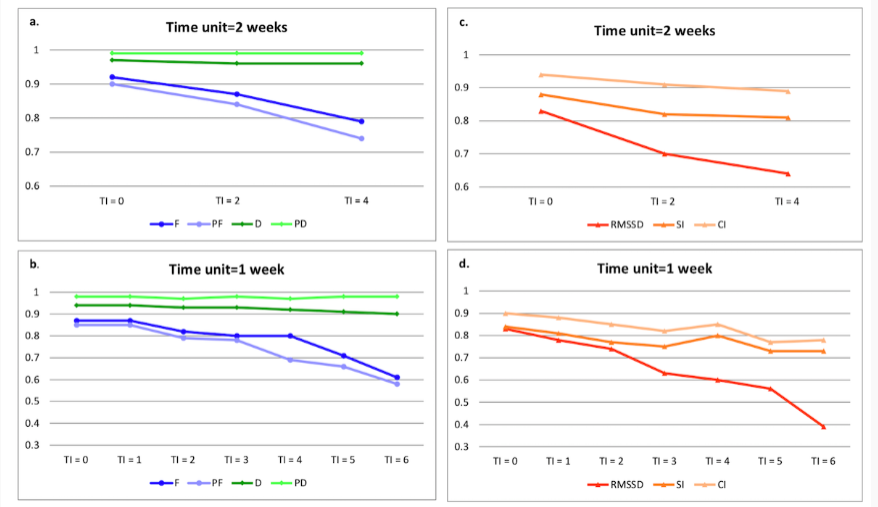
The temporal stability of app-generated parameters: Independent cycle. (a) The correlations of 2 independent 2-week uses with different time intervals for daily use frequency and duration parameters. (b) The correlations of 2 independent 1-week uses with different time intervals for daily use frequency and duration parameters. (c) The correlations of 2 independent 2-week-use periods with different time intervals for RMSSD, the SI, and the CI. (d) The correlations of 2 independent 1-week-use periods with different time intervals for RMSSD, the SI, and the CI. CI: Control Index; D: Total use duration; F: Total use frequency; PD: Proactive use duration; PF: Proactive use frequency; RMSSD: root mean square of the successive differences (between the adjacent duration of use and nonuse episodes); SI: Similarity Index; TI: Time interval (week).

Figure 4 shows the correlations of 2 independent 2-week uses with different TIs for daily use frequency and duration parameters. All correlations between 2 adjacent 2-week use periods (TI=0) for frequency and duration parameters are above .90. For 2 2-week use periods with a 2-week interval (TI=2), the correlations are all above .75. However, with regard to 2 2-week periods with 4-week intervals (TI=4), the correlation of proactive use frequency drops below .75. Figure 4 shows the correlations of 2 independent 1-week use periods with different TIs for daily use frequency and duration. All correlations between 2 1-week use periods with a TI less than 3 weeks (TI=0, 1, 2, and 3) are above .75. For 2 1-week use periods with a TI more than 4 weeks (TI=4, 5, and 6), the correlation of proactive use frequency drops below .75. The correlation of use frequency also drops below .75 when TI is more than 5 weeks (TI=5 and 6). It is worth noting that, for use duration

parameters, all correlations between 2 2-week use periods and 1-week use periods are all above .90, regardless how many TI s exist.

Figure 4 shows the correlations of 2 independent 2-week use periods with different TIs for RMSSD, the SI, and the CI. All correlations between 2 adjacent 2-week use periods (TI=0) are above .80. For 2 2-week-use periods with a 2-week interval (TI=2), the correlations of the SI and the CI are above .75 but not RMSSD. For 2 2-week use periods with a 4-week interval (TI=4), the correlation of RMSSD drops below .65. Figure 4 shows the correlations of 2 independent 1-week periods with different TIs for RMSSD, the SI, and the CI. For RMSSD, the correlations between 2 1-week-use periods with a TI more than 2 weeks (TI=2, 3, 4, 5, and 6) drop below .75 (*r*=.39, for TI=6). For the SI, the correlations between 2 1-week use periods drop below .75 when with a TI more than 4 weeks (TI=5 and 6). For the CI, all correlations between 2 1-week use periods are above .75, regardless of how many TIs exist.

## Discussion

### Principal Findings

This study adopted a 2-month period data to validate temporal stability of smartphone use. This study will enable researchers to construct a more structured methodology when collecting longitudinal behavioral data via the app. Most self-report scales assessing problematic mobile use also request the responders to report their smartphone usage within 3 months [10-12]. However, “digital phenotype” is a new concept referring to data generated passively from day-to-day interaction with a smartphone, it can provide real-time data on an individual’s environment and possibly the individual’s mental state [13]. Our findings highlight a crucial methodological issue on delineation of human behaviors by smartphone. If researchers want to investigate a 2-month period of smartphone use, a 2-week period of use data should be collected for consistency. In addition, when the TI between 2 smartphone use periods is more than 4 weeks, we should consider that these 2 periods belong to different use cycles, regardless of whether the use periods are for 1 week or 2 weeks.

### Strengths and Limitations

Our findings extend the previous work on tracking smartphone use pattern in several ways. First, a previous study had demonstrated that a relatively short duration of behavioral data (ie, 5 days) is required to qualify as a 2-week use period [5]. We assessed smartphone use for a longer time framework. Our previous studies have also found that smartphone use and nonuse patterns are reciprocal and have a cycle repeated weekly [7]. Therefore, a complete record comprising weekdays and weekends may be crucial to reflect typical smartphone usage. Even a 1-month record might not be enough to allow detection for patterns of smartphone use. In this study, we extended the app-recorded time frame to 2 months, which included at least 8 weekly cycles of smartphone use data. Our finding suggested that a 2-week smartphone use duration is an adequate fundamental time unit to infer a 2-month period of use, namely a record which accounts for 25% of the total use period may be sufficient. Investigating reliability such as temporal stability of measurements (ie, app-recorded parameters) is the very first step for collecting longitudinal data. Future studies examining smartphone use behaviors, self-reported variable relevant to smartphone use, and their interactions over time are urgently needed. Second, self-reported problematic smartphone use did not correlate with actual use recorded by an app in previous studies [4,5]. They concluded this may be because of the automatic nature of compulsive use and therefore cannot be captured through self-reported scales. However, we found a significant correlation between proactive use duration in 2 months and smartphone addiction. Self-reported smartphone addiction may correlate with long-term rather than short-term smartphone use. It may also imply that only proactive use duration correlates with self-reported smartphone addiction. Finally, we adopted more app-generated parameters than previous studies and evaluated their efficacy. The current findings support our previous study, which showed that not only use frequency and duration but also use/nonuse reciprocity is important when delineating smartphone use behaviors.

There are several implications of our findings related to the 3 series of app-generated parameters. Total use frequency and use duration are parameters that should be studied most often. Previous literature also demonstrated that self-reported total use duration is a risk factor of problematic smartphone use [14,15]. The parameters regarding total use may reflect a general pattern of human-device interaction. In this study, any smartphone use episode was recorded as screen-on to screen-off. It provides an opportunity to distinguish between proactive and reactive use. Our previous studies have found that proactive use is more relevant to the addictive behavior, whereas reactive use should be treated more like “signal noise” with regard to its passive nature [3]. In this study, only proactive use duration significantly correlated with self-reported smartphone addiction. Proactive use may be more important than checking behaviors following notifications as it reveals more information about intention, such as compulsive checking for messages or craving for a specific app. Furthermore, averaged daily RMSSD and SI have been applied to indicate impaired control for smartphone use in our previous study [3]. The use/nonuse parameters (ie, RMSSD, SI, and CI) give us a chance to assess the reciprocal patterns of smartphone use and may represent control ability of individuals. In this study, CI demonstrated better temporal stability than SI and RMSSD. The CI may be a more representative parameter to reflect smartphone users’ control ability.

There are several methodological limitations that should be noted. First, smartphone uses were defined by screen-on and screen-off. This definition cannot completely represent the status of smartphone use. Second, the study utilized a selected sample with excessive smartphone use (average daily smartphone use duration: 5.74 hours/day), which limits the ability to generalize these findings. Third, our sample size is small and from a convenience sample (85% of the sample is male). A larger sample size and nongender-biased sample are also needed for future study evaluating the efficacy of app-generated parameters in smartphone use. More transnational and cross-culture research is needed to validate the temporal stability of app-generated parameters in other countries. Fourth, we reported utility and temporal stability of use/nonuse parameters in this study as our previous works showed that these parameters were highly consistent with psychiatrists’ clinical diagnosis. A higher value of use/nonuse parameters represented lower use/nonuse similarity, and it was also associated with higher flexibility of smartphone use. However, different smartphone use patterns may still generate identical value on these parameters. For example, frequent, long use periods spread out in very even short intervals may generate similar CI with sparse use period with sporadic checking. It is still noteworthy that all the app-generated parameters that we introduced were only objective measurements used to delineate smartphone use patterns, and future studies are needed to elaborate their definition and utility.

### Conclusions

In conclusion, this study validated temporal stability for smartphone use patterns recorded by an app. Our findings suggest it is necessary to collect biweekly use data for evaluating smartphone use behaviors. In addition, the long-term smartphone use duration recorded objectively in a naturalistic setting is relevant to subjectively reported symptoms of smartphone addiction. The results may provide researchers an opportunity to investigate human behaviors longitudinally, using more structured methods via smartphone.

## Abbreviations

CI: Control Index
RMSSD: root mean square of the successive differences
SI: Similarity Index
SPAI-5: 5-item Smartphone Addiction Inventory
TI: time interval
